# Ecological shifts underlie parallels between ontogenetic and evolutionary allometries in parrotfishes

**DOI:** 10.1098/rspb.2024.1897

**Published:** 2024-10-30

**Authors:** Mayara P. Neves, April Hugi, Howan Chan, Kaleigh Arnold, Kara Titus, Mark W. Westneat, Miriam L. Zelditch, Simon Brandl, Kory M. Evans

**Affiliations:** ^1^ Department of Biosciences, Rice University, Houston, TX, USA; ^2^ Department of Organismal Biology and Anatomy, University of Chicago, Chicago, IL, USA; ^3^ Museum of Paleontology, University of Michigan, Ann Arbor, MI, USA; ^4^ Department of Marine Science, The University of Texas at Austin, Marine Science Institute, Port Aransas, TX 78373, USA

**Keywords:** Labridae, coral reef, development, allometry, evolution

## Abstract

During ontogeny, animals often undergo significant shape and size changes, coinciding with ecological shifts. This is evident in parrotfishes (Eupercaria: Labridae), which experience notable ecological shifts during development, transitioning from carnivorous diets as larvae and juveniles to herbivorous and omnivorous diets as adults, using robust beaks and skulls for feeding on coral skeletons and other hard substrates. These ontogenetic shifts mirror their evolutionary history, as parrotfishes are known to have evolved from carnivorous wrasse ancestors. Parallel shifts at ontogenetic and phylogenetic levels may have resulted in similar evolutionary and ontogenetic allometric trajectories within parrotfishes. To test this hypothesis, using micro-computed tomography (μCT) scanning and three-dimensional geometric morphometrics, we analyse the effects of size on the skull shape of the striped parrotfish *Scarus iseri* and compare its ontogenetic allometry to the evolutionary allometries of 57 parrotfishes and 162 non-parrotfish wrasses. The young *S. iseri* have skull shapes resembling non-parrotfish wrasses and grow towards typical adult parrotfish forms as they mature. There was a significant relationship between size and skull shapes and strong evidence for parallel ontogenetic and evolutionary slopes in parrotfishes. Our findings suggest that morphological changes associated with the ecological shift characterizing interspecific parrotfish evolution are conserved in their intraspecific ontogenies.

## 1. Introduction

During ontogeny, multicellular organisms commonly undergo significant changes in shape concomitant with increases in size. These alterations, crucial for immediate survival and long-term reproductive success, illustrate the remarkable adaptability of organisms to the functional demands of each life stage [1–3]. Recognizing the emergence of phenotypic diversity during development is pivotal for comprehending the role of developmental processes in the evolution of phenotypic traits.

Generally, allometry, defined as size-required change in shape [4], has been regarded as a constraint in evolution by representing a functional restriction, with size-related limitations on how the functions of distinct regions can change in tandem with shifts in body size, upholding functional equivalence across varying scales [5]. Still, allometry can act as an intrinsic restriction, as a paradigm embodying a spectrum of constraints, and plays a crucial role in morphological integration [6]. Furthermore, studies have suggested that allometry serves as an important factor influencing morphological diversity, as traits that would not be connected otherwise are observed to be correlated with body size [7]. Allometry can be represented in three different ways: static allometry, which involves comparing individuals of the same species and developmental stage [8]; ontogenetic allometry, which contrasts various developmental stages within a species and constitutes the initial definition of allometry [9]; and evolutionary allometry, which compares different species at the same developmental stage [10]. The complexities of evolutionary allometry extend beyond developmental origins and determinants, encompassing the dynamic interplay of size and shape in the evolutionary process [6].

The vertebrate skull is a dynamic and multi-functional structure during both development and evolution. Ontogenetic changes in skull shape during ecological shifts reflect the dynamic interplay between development and environmental demands [1,3,11,12]. These changes encompass modifications in size, shape and morphology of structures related to feeding that are seemingly tailored to the requirements of diverse environments [2,13]. Functional adaptations, such as adjustments in jaw mechanics and sensory features, contribute to the organism’s ability to thrive in its new ecological context [14,15]. Allometric growth patterns further influence the proportions of the skull relative to overall body size either in the maintenance of functional equivalence through changes in body size, or through the differential growth of structures as their respective functions change during ontogeny [16]. Ontogenetic changes in the function of a structure may correspond to ecological shifts as an organism changes diets, habitat or even its behaviour over time. Over extended periods, these ecological shifts may drive adaptive evolution, fostering genetic changes that confer advantages in the face of new environmental challenges [2,17]. Understanding these ontogenetic adaptations is crucial for revealing the ecological and evolutionary consequences of environmental changes in populations, contributing to a broader understanding of adaptive responses in various ecosystems.

Among the vertebrate clades that exhibit immense ecological and morphological diversity, the Labridae (Acanthomorpha: Eupercaria) stand out as an excellent model clade to study the contribution of allometry in an ontogenetic and evolutionary context. This diverse fish family, encompassing wrasses, weed whitings and parrotfishes, are common in coral reef ecosystems where they have evolved a myriad of adaptations to exploit different resources in their respective habitats. These adaptations include burrowing in the sand-diving wrasses [18,19], planktivory in both the hypsigenines and the cirrilabrines, complex cleaning behaviours in the julidines and perhaps most strikingly the ability to feed on hard substrates within parrotfishes, scarinines [20].

Parrotfishes predominantly occur on tropical coral reefs and are known for their vibrant colours, beak-like mouth structures and their vital role in coral reef functioning by removing primary producers, eroding calcareous surfaces and freeing up settlement space for benthic organisms like scleractinian corals [21–23]. These fishes also undergo notable ecological shifts during development, intricately linked to changes in diet and habitat preferences [21,24–27]. As larvae and recently settled juveniles (i.e. stage where juvenile individuals transition from drifting larvae to selecting a suitable habitat, such as coral reefs for growth and development), many parrotfish species possess individual teeth and feed primarily on invertebrates [27–29]. However, shortly after settlement, juveniles begin to coalesce their teeth into robust tooth plates in the upper and lower jaws and transition to feeding predominantly on algae, biofilms, sponges and dead corals, a diet that they maintain through adulthood [25,28,30]. The ecology and morphology of adult parrotfishes have been the subjects of extensive research spanning a century. However, there have been comparatively few studies of parrotfish ontogeny in a quantitative framework, and it is not clear how parrotfish beaks and skulls change and develop in parallel with the dietary shifts that they experience during growth [20]. The evolutionary history of parrotfishes adds further intrigue to this uncertainty. Parrotfishes are nested within the family Labridae [31] and thus evolved from wrasse ancestors, and this phylogenetic transition is accompanied by a distinct dietary shift from carnivorous diets to more herbivorous diets accompanied by changes in feeding strategies and jaw kinematics [20,27,32–35]. Parrotfishes display a wide range of trophic ecologies ranging from mostly herbivorous diets to more carnivorous diets in some of the smaller species [28,36–41]. These ecological shifts in diet during development and evolution may therefore coincide with allometric shifts in traits associated with feeding in parrotfishes. The conservation of these ecological shifts and the morphological changes associated with them at both the developmental and phylogenetic scale may result in parallel developmental and phylogenetic allometries.

We test this hypothesis using the ontogenetic skull shape allometry of the striped parrotfish, *Scarus iseri* (Bloch, 1789), combined with skull shape and phylogenetic datasets of 219 labrid species (wrasses and parrotfishes) to estimate evolutionary allometric trends. Our study seeks to address the following three important questions. (i) To what extent does allometry shape the developmental trajectory of the parrotfish skull including its individual bones? (ii) How do allometric trajectories evolve when an organism experiences an ecological shift, encompassing changes in habitat or diet, during both development and evolution? (iii) In the scenario where ecological shifts are conserved during development and evolution, can we anticipate parallelism in both developmental and evolutionary allometries? We hypothesize that we will recover a strong relationship between shape and size during ontogeny among the skull bones directly associated with feeding (e.g. premaxilla, dentary, maxilla, angular, lower pharyngeal tooth plate and the neurocranium) and that the ontogenetic allometry of *S. iseri* will parallel the evolutionary allometries that we observe among parrotfishes. This investigation is crucial for understanding the developmental adaptations of species and how they align with evolutionary patterns among parrotfishes, providing insights into the ecological and evolutionary mechanisms shaping feeding strategies.

## 2. Material and methods

### (a) Morphometric data collection and shape analyses

To investigate growth allometry, we examined the ontogenetic series of *S. iseri*, encompassing 54 individuals with a total length ranging from 1.75 to 33.5 cm. We chose this species because ontogeny plays an important role in the behaviours of *S. iseri* with resource, habitat and territory selection changing across life stages [28,29,42], and the species is generally abundant on Western Atlantic coral reefs. On these reefs, terminal phases (i.e. the later reproductively male stage of adult parrotfishes, characterized by vibrant and often striking coloration used for various purposes such as attracting mates, establishing dominance within the social hierarchy and signalling reproductive readiness) generally form mating territories that contain several schools of initial phases (i.e. early stage of adult reproductively female parrotfish) [30]. Although an individual mature territory contains several early-stage individuals, they are not found schooling and actively defend their territories against other mature individuals [30]. A majority of an adult’s time is spent defending their mating resources, but they will occasionally graze on sponges, corals, algae and sand [29]. Individuals in the early stages of life establish territories within the greater mature individuals’ territory but select their territory based on the availability of algal resources on a reef [30]. They tend these algal territories with other early-stage individuals while feeding on branching sponges, filamentous algae and sand [29]. Juvenile phase *S. iseri* do not establish territories and travel between patches of reef, mangroves and seagrass beds [42–44] often travelling in schools with other juvenile *S. iseri* as well as other species of parrotfish and wrasses [28,45,46].

For skull shape comparison analyses among *S. iseri*, other parrotfishes and other non-parrotfish wrasses, our sample of adults comprises 259 individuals (83 adult parrotfishes and 176 adult non-parrotfish wrasses) comprising 219 labrid species (electronic supplementary material, table S1). From this dataset, we compared the ontogenetic allometry of *S. iseri* to evolutionary allometry of Scarinines (57 species) and other non-parrotfish wrasses (162 species). The ontogenetic series of *S. iseri* was obtained through sampling carried out in Carrie Bow Caye, Belize in 2023, with additional individuals from the Field Museum of Natural History. The remaining species were obtained through loans from museum collections (electronic supplementary material, table S1).

We assessed the three-dimensional (3D) skull shape of each labrid species through micro-computed tomography (μCT) scans [47]. Specimens were scanned at the Rice University, the University of Texas Austin, the University of Minnesota, the University of Chicago and the University of Washington Friday Harbor Labs in conjunction with the scanAllFishes and oVert initiatives. The scans were segmented in Amira v2.0.0 (Thermo Fisher Scientific, Waltham, MA, USA) to isolate the skull while eliminating scales and debris. The isolated skulls were transformed into 3D meshes and imported into Checkpoint (Stratovan, Davis, CA, USA). We followed the landmark scheme of [20,34]; however, three additional landmarks detailing points along the pectoral girdle were incorporated [33]. The characterization of shape variation involved 200 3D points, 83 landmarks and 117 semi-landmarks. These points comprehensively sampled the pharyngeal jaws, oral jaws, neurocranium, nasals, hyomandibula, operculum, hyoid apparatus and pectoral girdle. All points were exclusively situated on the left side of the skull.

To accommodate for the rotation and translation inherent in highly kinetic articulating components of the fish skull, we initially aligned each dataset through generalized Procrustes analysis using the *gpagen* function in *geomorph* package, v. 4.0.4 [48] in R Statistical Environment, version 4.3.2 [49]. Subsequently, a local superimposition was conducted to standardize the positioning of diverse skull elements [50,51]. To analyse the shape evolution of individual bones, we partitioned our extensive skull shape dataset into smaller datasets for each bone, and the raw coordinates were individually superimposed. In this context, size was quantified as centroid size (CS), which represents the square root of the summed squared distances of each landmark to the centroid. After the local superimposition, we computed the average shapes and CSs for the adult specimens of each species.

### (b) Ontogenetic allometry of *S. iseri*

To visualize the shape variation of entire skull and individual bones of *S. iseri* across development, we used principal component analysis (PCA) using *gm.prcomp* function in *geomorph* package [48]. To estimate coefficients of ontogenetic allometry and test the effects of size on skull and bone shapes (superimposed coordinates), we used a Procrustes analysis of variance [52,53], fitting a model with one main effect, the factor ‘size’, measured as the natural logarithm-transformed centroid size (LCS). The Procrustes ANOVA was performed using the *procD.lm* function in *geomorph* [54,55]. To visualize the ontogenetic allometric patterns of *S. iseri* without considering groups, we used the *plotAllometry* function in *geomorph* with the method set to ‘RegScore’. This method computes standardized shape scores from the regression analysis of shape against size and then plots these scores relative to size [56].

### (c) Ontogenetic and evolutionary allometry comparisons

Initially, to visualize the variation in the shape of the entire skull and individual bones of *S. iseri* across development and contrast it with the shape of other parrotfishes (57 species) and other non-parrotfish wrasses (162 species), we used PCA [48].

To explore the evolutionary allometry of wrasses and parrotfishes, we used a recently published phylogenetic hypothesis involving 410 species of labrid fishes [34]. The tree was pruned down to include the 219 taxa for which the morphometric data were available, a process accomplished using the *drop.tip* function in the *ape* package v. 5.0 [57]. To derive allometric coefficients and assess the evolutionary allometry of parrotfishes (57 species) and other non-parrotfish wrasses (162 species), we employed a phylogenetic analysis of covariance (PGLS) [58,59]. The model encompassed one principal effect: the factor ‘size’, measured as the LCS. The phylogenetic ANCOVA was fitted using the *procD.pgls* function in *geomorph*. We used a Type II sums of squares analysis for statistical tests of model terms [59]. In line with the analysis of growth allometry, tests of statistical significance were conducted through permutations of the residuals from the model. The statistical significance of the slopes was determined by comparing the angles to the distribution of random values. Afterwards, pairwise comparisons between ontogenetic and evolutionary allometries were conducted using the *pairwise* function in residual randomization in permutation procedures (RRPP) package [55]. Beyond assessing the null hypothesis that the angles differ no more than expected by chance, we also examined the hypothesis that angles are no more similar than anticipated by chance.

In phylogenetic comparative methodological approaches that utilize regression models, it is necessary to adjust the covariance matrix of the error term to account for phylogenetic non-independence. Traditionally in phylogenetic ANOVAs, the underlying covariance error structure is that of a Brownian motion model. However, a Brownian motion process may not always adequately fit the data, leading to inaccurate estimates, especially for estimating allometric slopes. Pagel’s lambda offers an alternative approach to transforming the covariance matrix [6]. This transformation involves rescaling the branch lengths of the phylogenetic tree so that they result in expected constant variances on the transformed scale. By doing so, we can account for the phylogenetic signal in the residuals, which ideally should be estimated simultaneously with the regression parameters. However, in the case of high dimensional shape data, directly estimating the phylogenetic signal along with regression parameters can be computationally challenging [6].

To address this, we used a methodological compromise. We first reduced the ordinary least squares (OLSs) residuals [58,59] using PCA to reduce the dimensionality of our data. Then, we estimated Pagel’s lambda for the reduced multivariate datasets (i.e. evolutionary skull shape dataset and individual bone dataset). Finally, we transformed the phylogeny using the estimated lambda value. This process helps to adjust for phylogenetic signal in the residuals, ensuring that the subsequent analyses provide more accurate estimates while accounting for the evolutionary relationships among species. For our shape evolution dataset, we estimated Pagel’s *λ* utilizing the *transformPhylo.ML* function in the *motmot* package v. 2.1.3 [60]. Then, we transformed the phylogeny by the *λ* value using the *rescale* function in the *geiger* package version 2.07 [61].

Finally, to compare the ontogeny of *S. iseri* and the evolutionary allometry of parrotfishes and other non-parrotfish wrasses, we conducted pairwise analyses between vectors of allometric coefficients (i.e. the coefficients of the size and shape), the growth allometries estimated by OLS and evolutionary allometries estimated by PGLS. Following the methodology outlined by [6], we executed pairwise comparisons by extracting fitted values (i.e. predicted values for all individuals) from the OLS and PGLS models. This approach was found to yield more accurate estimates of the upper bound of the confidence interval than the alternative: predicting the shapes at the smallest and largest sizes and then adding the residuals of the models to those endpoints [62,63]. These values were then combined, and a subsequent ANCOVA was performed using the *procD.lm* function in the *geomorph* package. This ANCOVA involved regressing the fitted values against size and comparing the ontogeny and evolutionary allometry (i.e. ontogeny of *S. iseri* versus evolutionary allometry of parrotfishes, and ontogeny of *S. iseri* and versus evolutionary allometry of all labrids). Pairwise comparisons between vectors were carried out, utilizing the *pairwise* function in RRPP [55]. To visualize the ontogenetic trajectory of *S. iseri* and evolutionary allometric trajectories of parrotfishes and other non-parrotfish wrasses, we also used the *plotAllometry* function in *geomorph* with the method set to ‘PredLine’. ‘PredLine’ method takes the multivariate matrix of predicted values from the regression and extracts the first principal component of the fitted values, plotting them against size [64].

## 3. Results

### (a) Cranial morphology of *S. iseri* across ontogeny

**Figure 1.**
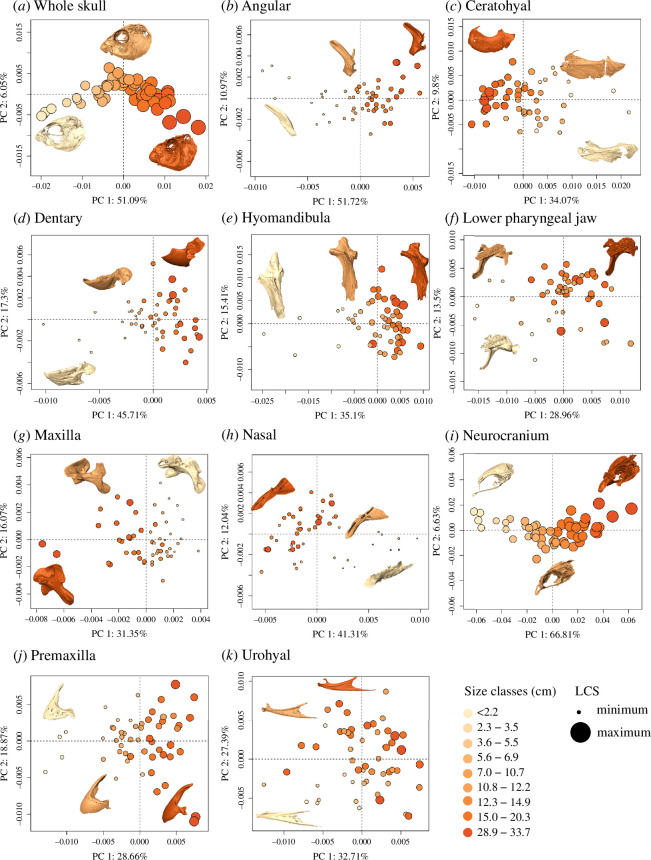
Variation in (*a*) skull and (*b*–*k*) bone shapes across ontogeny in *S. iseri* classified by total length classes.

**Figure 2.**
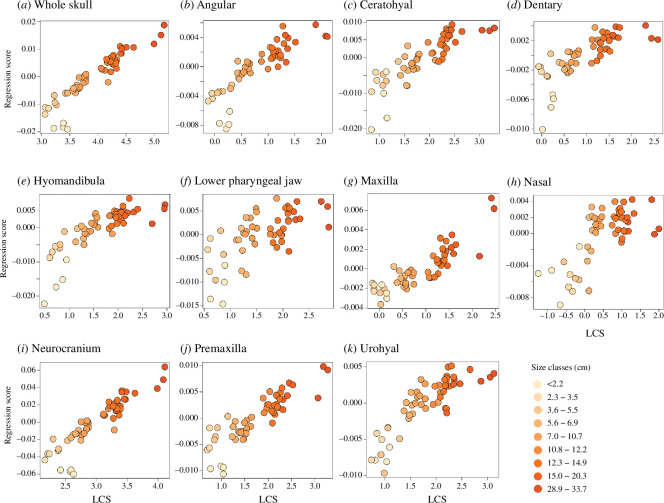
Allometric effects on skull (a) and bone shape (b-k) development of *S. iseri*.

Size has a variable effect on the development of skull and bone shapes in *S. iseri* (figure 1; table 1). The most conspicuous changes were observed in the overall skull shape, neurocranium and angular bones, which display curvilinear trajectories. The smallest individuals exhibited shallow, slightly elongated skulls, large eye sockets and lack of fully coalesced beaks; instead, they exhibited individual teeth (electronic supplementary material, figure S1). Notably, adults, particularly those surpassing a total length of 10.8 cm, exhibited shorter and wider skulls with posteriorly displaced, relatively contracted orbits, deep dentaries and greatly expanded supraoccipital crests of the neurocranium. Concurrently, the angular and dentary bones exhibited a trend of decreasing in relative length and became more truncated with size. Similarly, with a complete formation of the beak, the premaxilla became shorter with the relative contraction of the ascending process and the expansion on the tooth plate. The ceratohyal, dentary, hyomandibula and premaxillary bones displayed more significant variation among individuals smaller than 2.2 cm, but similarities emerged in later size classes. The effects of growth on urohyal and the lower pharyngeal jaw bones were less pronounced. However, strong allometric effects were observed in the overall skull and individual skull bones (table 1; figure 2).

**Table 1.** Effects of ontogenetic allometry on the whole skull and individual bones of *S. iseri*. SS denotes the standard deviations of observed sum of squares values from sampling distributions of random values. MS denotes mean square.

		Df	SS	MS	Rsq	*F*	*Z*	*p*
whole skull	LCS	1	0.004	0.004	0.424	38.324	3.018	0.001
	residuals	52	0.005	0.000	0.576			
	total	53	0.009					
angular	LCS	1	0.000	0.000	0.364	29.796	4.613	0.001
	residuals	52	0.001	0.000	0.636			
	total	53	0.001					
ceratohyal	LCS	1	0.003	0.003	0.270	19.264	3.927	0.001
	residuals	52	0.008	0.000	0.730			
	total	53	0.011					
dentary	LCS	1	0.000	0.000	0.265	18.701	5.028	0.001
	residuals	52	0.001	0.000	0.735			
	total	53	0.001					
hyomandibula	LCS	1	0.001	0.001	0.206	13.508	5.390	0.001
	residuals	52	0.005	0.000	0.794			
	total	53	0.006					
lower pharyngeal jaw	LCS	1	0.001	0.001	0.109	6.364	4.183	0.001
	residuals	52	0.005	0.000	0.891			
	total	53	0.006					
maxilla	LCS	1	0.000	0.000	0.229	15.409	6.622	0.001
	residuals	52	0.001	0.000	0.771			
	total	53	0.001					
nasal	LCS	1	0.000	0.000	0.177	11.192	4.218	0.001
	residuals	52	0.001	0.000	0.823			
	total	53	0.002					
neurocranium	LCS	1	0.035	0.035	0.541	61.166	3.499	0.001
	residuals	52	0.030	0.001	0.460			
	total	53	0.065					
premaxilla	LCS	1	0.001	0.001	0.209	13.753	5.851	0.001
	residuals	52	0.003	0.000	0.791			
	Total	53	0.004					
urohyal	LCS	1	0.001	0.001	0.174	10.954	4.844	0.001
	residuals	52	0.003	0.000	0.826			
	total	53	0.003					

**Figure 3.**
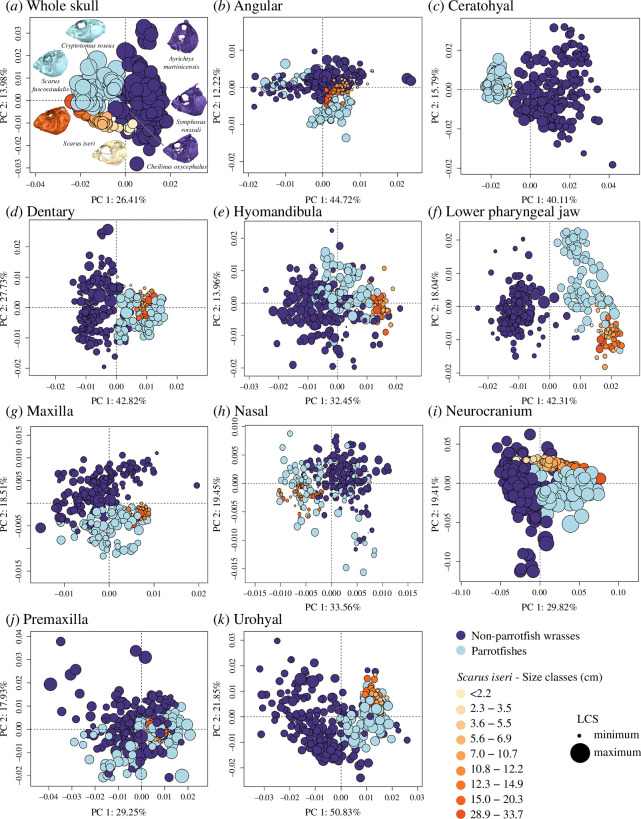
Shape variation of the (*a*) skull and (*b*–*k*) individual bones of *S. iseri* (orange) across ontogeny. The smallest individuals usually resemble their non-parrotfish wrasses relatives (purple) while the adult individuals resemble other adult parrotfishes (light blue).

When comparing the ontogenetic trajectory of *S. iseri* to our evolutionary shape dataset, we find that juvenile *S. iseri* (<5.5 cm total length) more closely resemble non-parrotfish wrasses, and specifically cheilinine wrasses, than other adult parrotfishes across the whole skull shape dataset and across the angular, ceratohyal, dentary and neurocranium. These juveniles notably exhibit a narrow and elongated skull with a large, anteriorly displaced orbits, an extended ascending process of the premaxilla and the presence of uncoalesced teeth (figure 3; electronic supplementary material, figure S1). Throughout ontogeny, the skull shape progressively converges towards the morphology observed in other adult parrotfish species. These morphological changes are primarily concentrated in the neurocranium, orbit, nasal, dentary and premaxilla (figure 3). Conversely, other skeletal elements maintain a consistent shape across ontogenetic stages, resembling the morphology found in other adult parrotfish species. Notably, juveniles and adults share similar shapes in the lower pharyngeal jaw, maxilla and urohyal.

### (b) Ontogenetic and evolutionary allometry comparisons

**Figure 4.**
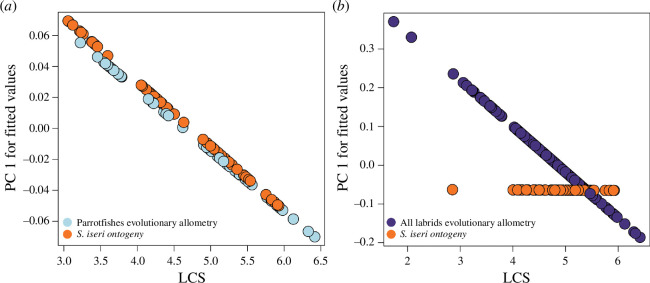
Effects of ontogenetic allometry on the skull shape of *S. iseri* (54 individuals) and evolutionary allometry on the skull shape of (*a*) parrotfishes (Scarinines: 57 species) and (*b*) all labrids (wrasses and parrotfishes: 219 species). PC1 fitted values of evolutionary allometry plotted against the LCS. For statistical results and *p*-values, see table 3.

As predicted, the ontogenetic allometry of *S. iseri* and evolutionary allometry of parrotfishes were not statistically different, suggesting conservation between ontogenetic and evolutionary allometries (table 2, electronic supplementary material, table S2). However, there was a significant difference between the ontogenetic trajectory of *S. iseri* and evolutionary allometry of all labrids (table 2; electronic supplementary material, table S3). There was strong evidence that size (LCS) had a significant effect on the shape of the skull and its constituent bones of *S. iseri*, other parrotfishes and all labrids (table 3; electronic supplementary material; tables S4-S5). Plotting PC1 of the predicted regression values in relation to size reveals the component of allometry along PC1 and generally indicates that shape and size evolve in concert (figure 4). As size increases, the whole skull and neurocranium tend to become more truncated. For the other bones, size had a positive relationship with both ontogenetic and evolutionary shape change, indicating that as the fish grows, the size of the bones also increases (electronic supplementary material, figures S2–S3).

**Table 2.** Comparisons between ontogenetic allometry of whole skull of *S. iseri* (54 individuals), evolutionary allometry of parrotfishes (Scarinines: 57 species) and all labrids (wrasses and parrotfishes: 219 species). UCL denotes upper confidence limit.

group	*r*	angle	UCL (95%)	*Z*	Pr > angle
scarinines × *S. iseri*	0.999	2.384	4.439	0.355	0.375
all labrids (wrasses and parrotfishes) × *S. iseri*	0.134	82.308	2.069	8.551	0.001

**Table 3.** Effects of ontogenetic allometry on the whole skull of *S. iseri* and evolutionary allometry on the skull shapes of parrotfishes and all labrids. Group: *S. iseri* ontogeny (54 individuals), Scarinines (57 species) and all labrids (wrasses and parrotfishes: 219 species). For visualization, see figure 4. Abbreviations as for table 1.

group		Df	SS	MS	Rsq	*F*	*Z*	Pr(>*F*)
scarinines × *S. iseri*	LCS	1	0.098	0.098	0.594	165.462	5.666	0.001
	group	1	0.002	0.002	0.012	3.4	1.511	0.069
	LCS:Group	1	0.002	0.002	0.01	2.668	1.412	0.082
	residuals	107	0.063	0.001	0.384			
	total	110	0.165					
all labrids (wrasses and parrotfishes) × *S. iseri*	LCS	1	1.579	1.579	0.343	158.495	5.671	0.001
	group	1	0.142	0.142	0.031	14.267	2.754	0.003
	LCS:Group	1	0.198	0.198	0.043	19.907	3.379	0.001
	residuals	269	2.680	0.010	0.583			
	total	272	4.599					

## 4. Discussion

### (a) Evolutionary allometry is conserved through ontogeny

Allometry plays an important role in shaping patterns of morphological diversity and maintaining functional relationships across different traits concomitant with changes in size through development and across species [5–10]. As a result, allometric relationships can either be highly conserved because of functional constraints or themselves be the targets of natural selection for functional optimization as organisms and clades functionally diversify [65]. In this study, we tested for conservation between ontogenetic and evolutionary allometric slopes within a single parrotfish species and across the family Labridae, which undergoes significant dietary and morphological changes during development and represents a larger clade of fishes that have notably diverged from their ancestral phenotype in both form and ecology. We identified parallel ontogenetic and evolutionary allometric slopes, which are likely linked to parallel ecological shifts associated with diet, where during development, larval *S. iseri* exhibit carnivory and transition to durophagy and the consumption of primary producers. This parallels the dietary evolution of parrotfishes where ancestral parrotfishes transitioned from carnivorous diets to the primarily microphagous and herbivorous diets that they are known for today [20]. Notably, the youngest *S. iseri* individuals exhibit skull shapes more reminiscent of their non-parrotfish wrasse relatives, gradually transforming into the characteristic shape of adult parrotfish as they matured. The striking similarity of juvenile *S. iseri* skulls to the cheiline wrasse skull appears to be a hallmark of the close phylogenetic sister-group relationship between cheinines and scarines [31] and suggests that there are important developmental morphological traits that are retained from this close relationship. Interestingly, in a similar area of shape space as the juvenile *S. iseri*, we also find the bluelip parrotfish (*Cryptotomus roseus*) which is the smallest parrotfish species, which lacks a coalesced beak and exhibits a more carnivorous diet [26]. Beyond skull shape, *C. roseus* also exhibits a suite of traits that are not observed in other parrotfishes but are instead present in other wrasse species such as the possession of an elongate slender body and the ability to burrow into the sand to escape predators, a behaviour that was otherwise lost in parrotfishes [18,20,66]. This suggests that *C. roseus* may exhibit a paedomorphic skull shape phenotype and trophic ecology relative to other parrotfishes, further indicating body size as a major axis for phenotypic and functional diversification in parrotfishes.

### (b) Mosaic patterns of ontogenetic allometry across the parrotfish skull

Body size is widely considered to represent a ‘line of least resistance’ for trait evolution [67]. As a result, traits that covary strongly with body size may secondarily display wider ranges of morphological variation and diversity. However, different traits often exhibit different scaling relationships with body size across an organism during development. This mosaic pattern of ontogenetic allometry may influence downstream patterns of trait evolution as traits that covary less with body size may exhibit a lower range of variation in development and subsequently less diversity at the macroevolutionary scale [68]. In our analysis, not all skull constituent bones had similar ontogenetic trajectories; certain bones, such as the neurocranium and angular bone, displayed more noticeable changes in shape while lower pharyngeal jaws were more conserved across development. This mosaic pattern of allometric scaling likely substantially influenced downstream patterns of morphological evolution in labrid fishes as the neurocranium has been shown to exhibit the fastest rates of shape evolution within the skull, while the pharyngeal jaws evolve significantly slower [34]. More broadly, the pharyngeal jaw bones in fishes develop under strong *Hox* gene control and ossify earlier than most of the other regions of the skull during development [69,70]. The weak scaling relationship between pharyngeal jaw shape and body size that we observe in our study, coupled with the reduced rates of shape evolution in this region from previous studies, may indicate a potential developmental constraint that reduces that range of available phenotypic variation in the pharyngeal jaw region of fishes more broadly.

### (c) Skull shape ontogenetic shifts reflect changes in functional demands

Discrepancies between ontogenetic and evolutionary allometries are common across vertebrates [6,10,71]. However, ontogenetic changes in shape can also mirror evolutionary changes as observed between *S. iseri* ontogeny and evolutionary allometry of Scarinines. When evolutionary changes in shape parallel ontogenetic changes, it suggests that the functional demands driving these changes are similar. In other words, the same functional demands that influence an organism’s shape during its growth (ontogeny) may also drive shape changes over evolutionary time. Our results contrast sharply with the idea that such parallelism arises from intrinsic developmental constraints. Instead, it is the parallelism in functional demands that accounts for these observations, as supported by some recent studies [72–75].

The configuration and shape of the vertebrate skull are shaped by functional and ecological demands, rendering it a remarkably versatile and diverse structure [3]. The developmental stages of parrotfishes encompass a sequence of delicate phases, with pivotal moments of morphological changes and dietary shifts. In our study, *S. iseri* exhibited significant changes in skull shape morphology across its ontogeny, especially associated with the neurocranium, orbit size and beak formation. These structures can be associated with swimming efficiency, visual acuity and feeding [76]. Throughout ontogeny, the neurocranium becomes more contracted and truncated with the orbit posteriorly displaced as the fish matures. The posterior displacement of the orbit during development may be an adaptation for increased grazing activity because it allows for fishes to detect predators while feeding on exposed benthic substrates and has been reported in other herbivorous reef fishes [77]. In juvenile specimens of *S. iseri*, the orbit to skull ratio was found to be highest suggesting that smaller fishes possessed relatively large eyes compared to adults. This can be attributed to the fact that the rod and cone cells within the eyes of smaller bodies tend to be larger. Consequently, the eye must increase in size to accommodate an adequate number of cells within the eyeball [78]. In the jaw region, beaks coalesced as individuals increased in body size, likely closely tracking known shifts in diet. A fully formed beak allows larger individuals to access hard substrates resulting in increased foraging on epi- and endolithic primary producers and microbial decomposers [29] whereas juveniles without fully formed beaks and smaller jaw muscles (<3.5 cm) must forage on other substrate types that are more accessible for weaker bite forces and smaller jaw openings [79,80]. Moreover, in the initial developmental phases characterized by size constraints, adopting a generalist and carnivorous behaviour can provide numerous benefits to the individual [13]. This becomes particularly crucial during the early stages of life, identified as the period of utmost vulnerability and mortality for young fishes [81]. Feeding on energetically advantageous prey becomes beneficial for juveniles to survive through this initial phase and progress successfully to adulthood [13,76].

## 5. Conclusion

The present study represents a comprehensive effort to quantify and delineate the ontogenetic changes in skull shape for parrotfishes. It emphasizes a notable parallel between morphological alterations and shifts in ecological dynamics. Furthermore, our research compares the trajectories of ontogenetic and evolutionary allometry within the parrotfishes and wrasses at large. The observed allometric changes in skull shape appear to manifest gradually with an increase in size, with each constituent bone of the skull undergoing varied modifications. The findings suggest parallel evolution between ontogenetic and evolutionary allometry trajectories likely due to similar ecological shifts that occurred both during the evolution and development of parrotfishes. Importantly, our research emphasizes the necessity of employing quantitative multivariate analyses to gain a nuanced understanding of how developmental processes contribute to shaping phenotypic diversity across species.

## Data Availability

Data and R script are available from the Dryad Digital Repository [82]. Supplementary material is available online [83].
